# Evaluation of a new device for emergency transcricoid ventilation in a manikin model

**DOI:** 10.1186/cc12102

**Published:** 2013-03-19

**Authors:** P Persona, P Diana, A Ballin, F Baratto, M Micaglio, C Ori

**Affiliations:** 1 Clinica di Anestesia e Medicina Intensiva, Padova, Italy

## Introduction

Failed airway situations are potentially catastrophic events and require a correct approach with appropriate tools. Recently, Ventrain has been presented as a manual device for emergency ventilation through a small-bore cannula, which can provide expiratory assistance by applying the Venturi effect.

## Methods

We used the SimulARTI Human Patient Simulator to evaluate Ventrain. Initially, we studied the effectiveness and security in ventilating and oxygenating the patient. In a second phase, the Ventrain performance was compared with what is considered to be the present gold standard (Quicktrach II, Portex Mini-Trach II Seldinger Kit, Melker Emergency Cricothyrotomy Catheter Set). Seven anesthesiologists performed an emergency transcricoid ventilation with each device in the same setting.

## Results

**Figure 1:**
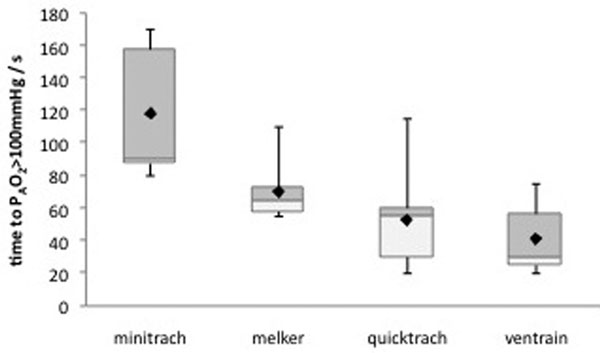
**Oxygenation time to oxygen alveolar pressure >100 mmHg**.

**Figure 2:**
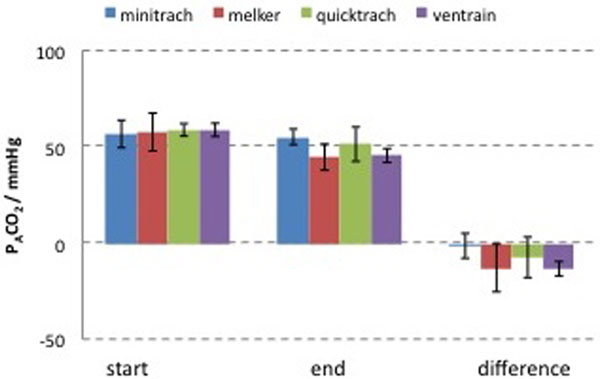
**Mean carbon dioxide alveolar pressure at the start and at the end of the test**.

Ventrain provided an average tidal volume of 334 ml and an average minute volume of 2.4 l in the considered situation, with a modification of PAO_2 _from 32 to 702 mmHg and of PACO_2 _from 54.5 to 38.8 mmHg. In the second phase, the time needed to obtain an effective oxygenation with Ventrain was found to be shorter than other devices (median difference; vs. Minitrach -60 seconds; vs. Melker -35 seconds; vs. Quicktrach -25 seconds) (Figure 1); the ability to remove CO_2 _resulted bigger (average difference: vs. Minitrach -11.9; vs. Melker -0.3; vs. Quicktrach -5.9) (Figure 2) and moreover the users judged it more favorably.

## Conclusion

In this manikin study, Ventrain seemed to be able to appropriately oxygenate and ventilate a patient in a CICV situation. When compared with the best available choices, it has shown not to be inferior.
